# The impact of empathy on medical students: an integrative review

**DOI:** 10.1186/s12909-024-05448-5

**Published:** 2024-04-25

**Authors:** Hao Chen, Hanwen Xuan, Jinquan Cai, Meichen Liu, Lei Shi

**Affiliations:** 1 Department of Neurosurgery, the Second Affiliated Hospital of Harbin Medical University, Harbin, 150086 China; 2 Modern Educational Technology Center, Harbin Medical University, Harbin, 150086 China; 3 School of Health Management, Guangzhou Medical University, Guangzhou, 511436 China; 4 School of Health Management, Southern Medical University, Guangzhou, 510515 China

**Keywords:** Empathy, Medical students, Mental Health, Clinical competence, Academic performance

## Abstract

**Introduction:**

Empathy is considered the ability to understand or feel others emotions or experiences. As an important part of medical education, empathy can affect medical students in many ways. It is still lacking a comprehensive evaluation of the existing articles on empathy’s impact on medical students, despite the existence of many articles on the topic.

**Objectives:**

To summarize the impact of empathy on medical students during medical education from four perspectives: mental health, academic performance, clinical competence, and specialty preference.

**Methods:**

The search terms used for retrieval were “empathy”, “medical student”, “mental health”, “depression”, “anxiety”, “burnout”, “examinations”, “academic performance”, “clinical competence”, “specialty preference” on PubMed, EBSCO, and Web of Science before January 2024. The search was carried out by two reviewers. Titles and abstracts were screened independently and reviewed based on inclusion/exclusion criteria. A consensus was drawn on which articles were included.

**Results:**

Our results indicated that high empathy was a positive factor for mental health, However, students with high affective empathy were more likely to suffer from depression, anxiety, and burnout. Empathy was found to be unrelated to academic performance, but positively correlated with clinical competence, particularly in terms of communication skills. Medical students with high levels of empathy tended to prefer people-oriented majors.

**Conclusions:**

Medical students who score higher on the self-reported empathy scales often have better mental health, better communication skills, and tend to choose people-oriented specialties. But empathy is not related to academic performance. Additionally, the different dimensions of empathy have different impacts on medical students. It is necessary to design targeted courses and training for medical students to enhance their empathy.

**Supplementary Information:**

The online version contains supplementary material available at 10.1186/s12909-024-05448-5.

## Introduction

The definition of empathy encompasses a wide range of social, cognitive, and affective processes, primarily involving the capacity to understand or feel the emotions or experiences of others [1]. Specifically, empathy enables us to resonate with the positive or negative emotions of others, meaning that when we indirectly share the happiness or pain of others, we also feel happy or painful. And due to empathy, people still know that the reason for their resonant feelings are the feelings of others, and do not confuse themselves with the feeling of others [2]. Furthermore, empathy is regarded as a multidimensional construct [3], comprising two main components: affective and cognitive empathy [4, 5]. Among them, affective empathy is defined as the capacity to respond emotionally to the mental states of others, which can be divided into empathic concern and personal distress [6]. While, cognitive empathy is a kind of capacity to understand the views or mental states of others [7], and can be subdivided into perspective-taking and fantasy. In addition, both congenital and acquired factors can influence an individual’s level of empathy [8].

From a medical perspective, empathy refers to the ability to understand and empathize with patients’ situations, perspectives, and feelings, enabling healthcare professionals to communicate effectively and provide treatment with the patients’ consent and assistance [9]. Therefore, empathy is a highly desirable trait in physicians, serving as a crucial component of the physician-patient relationship. It significantly influences both the diagnostic process and the delivery of treatment [10]. Patients who feel treated with empathy are more likely to fully explain their symptoms, provide relevant details, and actively engage in the patient-physician relationship [3, 11]. And, the empathy of physicians has positive impacts on gaining the trust of patients [12], effective communication [13], patient’s insistence on treatment [11], adherence to medical recommendations [14, 15], patient satisfaction [16–18], alleviating patient pain [19], improving treatment outcome [20, 21], and reducing potential legal risks [22, 23]. Empathy, in addition to its beneficial effects on patients, also has a profound impact on doctors and medical students, as it is closely tied to the well-being of physicians [24]. Doctors with lower levels of empathy are more likely to exhibit symptoms of stress and fatigue compared to their colleagues with higher levels of empathy [25]. Doctors with lower levels of empathy also experience higher levels of burnout and are more likely to be involved in medical accidents [26]. In addition, empathy is also related to the professional satisfaction of doctors [27]. All in all, empathy is beneficial for both patients and physicians, as emphasized by the General Medical Council in the United Kingdom [28] and the Association of American Medical Colleges [29] who have both underscored its importance in medical education for medical students. Besides, in order to provide high-quality patient care, the cultivation of medical affective should focus on both training medical skills and cultivating affective skills. Compared to clinical knowledge and skills, professional spirit and empathy are relatively more difficult to cultivate [30]. Considering that medical student, who officially become doctor after graduating from medical school, will constantly face emotional challenges such as pain, fear, and despair, cultivating and strengthening empathy is an important goal of medical education, aiming to prevent them from losing the ability to act professionally and ethically [31]. And a systematic review has shown that educational interventions can effectively maintain and enhance medical students’ empathy [32].

To assess individual empathy, researchers have developed a variety of scale tools, with the Jefferson Scale of Physician Empathy (JSPE) being a common measurement tool [33]. JSPE was originally developed to measure the empathy of medical students in patient care scenarios. In some versions, it is also referred to as the JSE. When compared to JSPE, the Interpersonal Reactivity Index (IRI) exhibits numerous differences in terms of its applicable population, measurement standards, and the conceptual and structural understanding of empathy. Specifically, IRI was developed for the general public, whereas JSPE was based on empathy in clinical settings and was developed for students and healthcare professionals. In addition, while the authors of IRI conceptualized empathy as a combination of cognitive and affective attributes, the authors of JSPE defined empathy as a cognitive attribute. These differences were reflected in the content of the project [34, 35]. Additionally, Baron-Cohen et al. proposed the Empathy Quotient, a tool designed to measure and quantify differences in empathy among individuals [36]. There are other measuring tools, such as the Questionnaire of Cognitive and Affective Empathy (QCAE) [37] and the Toronto Empathy Questionnaire, which are mostly used to measure the empathy of the general population [38].

Recently, Costa et al. reported that medical students’ empathy remained relatively stable throughout their medical education [39]. However, most studies examining the variation of empathy among medical students have demonstrated a tendency for empathy to decline during their medical school training. These researches originated from different countries, each possessing unique cultural systems, rather than being limited to a handful of nation [40–61]. And, in most studies, gender has been found to be a predictor medical students’ empathy levels, with females typically exhibiting higher levels of empathy than males [62, 63]. More importantly, empathy is believed to significantly impact various aspects of medical students’ educational experience. Researchers in this field have studied the importance of empathy in the educational process and discovered that different dimensions of empathy exert distinct influences on students’ mental health, academic performance, clinical competence, and specialty preferences.

Although evidence on the impact of empathy on medical students is increasing, it should be noted that most studies examining the role empathy played in the development of medical students have not been comprehensive. For example, the researches of Thomas et al., and Carrard et al., studied the impact of empathy on the mental health among medical students [64, 65]. The study of Javaeed et al., investigated the impact of empathy on the academic performance [66]. A study discussed the impact of empathy on the clinical competence [67], while another study from Iran involved the impact of empathy on the academic performance and specialty preference [68]. It should be noted that most studies examining the role empathy played in the development of medical students were not comprehensive. Each individual study only involved one or two aspects about the impact of empathy on medical students. At present, there is a lack of comprehensive evaluation of the overall impact of empathy on medical students. Furthermore, some researchers considered empathy as a single construct [33, 42, 47, 52, 62, 68–75], ignoring its multiple dimensions.

Therefore, the research question of this integrative review is as follows:

How does empathy impact medical students’ mental health, academic performance, clinical competence, and specialty preferences?

## Methods

We chose integrative review as the methodology to identify and synthesize the impact of empathy on medical students from literature. Integrative review allows researchers to combine experimental and non-experimental studies to fully understand theoretical aspects of the phenomenon analyzed. It also combines data from theoretical and empirical literature, and has a wide range of purposes, such as definition of concepts, review of theories and evidence, and analysis of methodological problems of a particular topic [76, 77]. While systematic review is an exacting synthesis of all investigations related to one specific question, focusing primarily on quantitative experimental studies, such as randomized clinical trials. It aims at overcoming possible biases in each stage, following a strict method to search and select investigations, assessing relevance and validity of the studies found [78]. Therefore, in order to extensively collect relevant references on the impact of empathy on medical students, we chose integrative review instead of a systematic review. We conducted this integrative review, using the methodological framework proposed by Whittemore and Knafl [76]. It involves the following stages: Problem identification, Literature search, Data evaluation, Data analysis and Presentation. Applied to this integrative review, each of these stages involved the following:

### Problem identification

The primary problem that guided this review was “How does empathy influence medical students’ mental health, academic performance, clinical competence, and specialty preferences?”

### Literature search

**Fig. 1:**
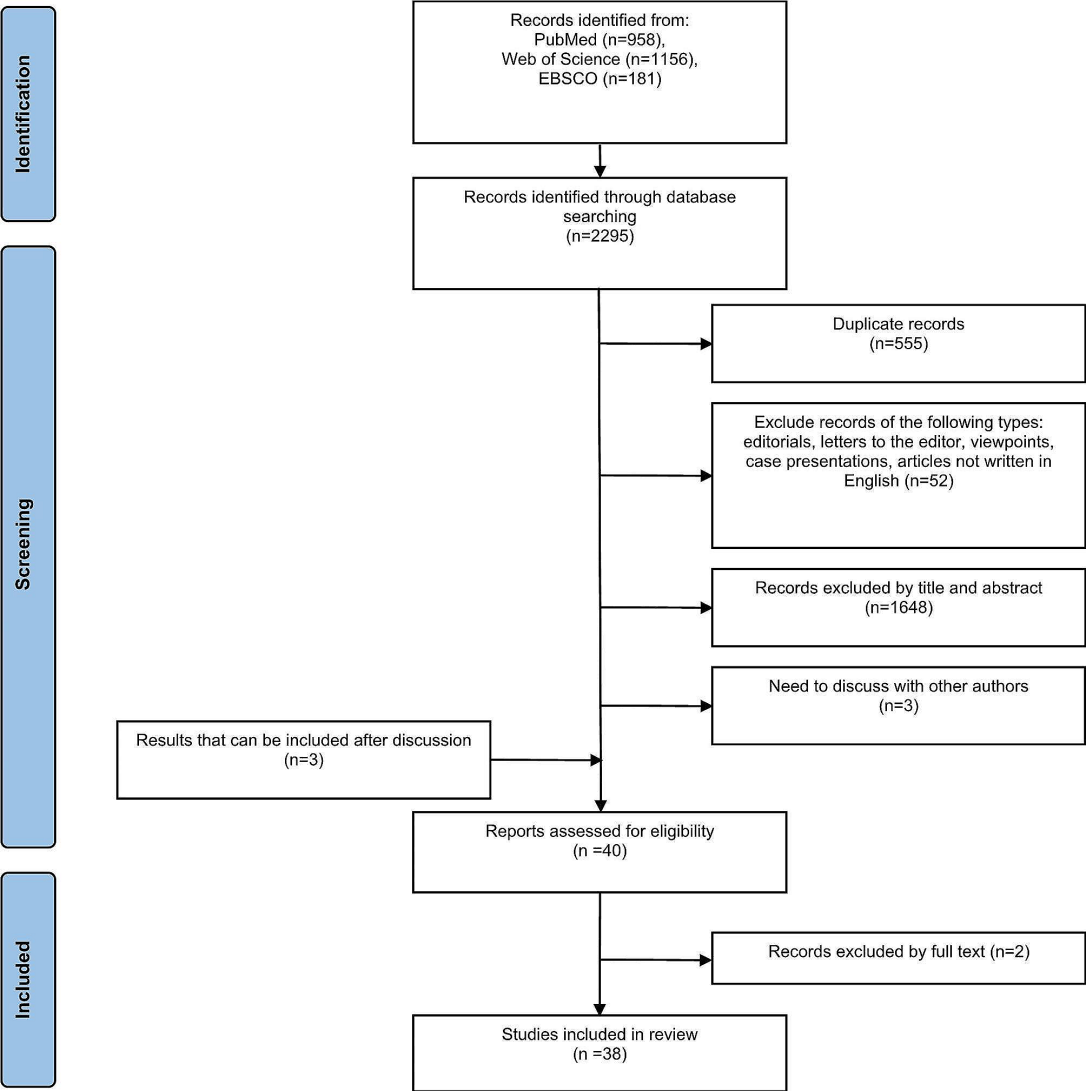
Flowchart of search and selection process

A search strategy was devised with input from the research team which was comprised of all authors. Two authors searched for articles on PubMed, EBSCO and Web of Science. The key search terms were “(empathy OR empathic OR empathetic) AND (medical student) AND (mental health OR depression OR anxiety OR burnout OR examination OR academic performance OR clinical competence OR specialty preference)”. A search process flowchart is shown in Fig. 1.

### Data evaluation

We formulated the selection criteria for this review by combining the research question and the inclusion/exclusion criteria of the previous integrative reviews [79–84]. The language used in the article, the research object, the empathy scale involved in the research and the purpose of the research were taken as the main evaluation indicators. Regardless of the publication date and journal, above two authors independently screened articles through referring to the titles and abstracts based on the predefined inclusion criteria. If necessary, they would read the entire text to assess whether the candidate article met the inclusion criteria. Subsequently, they independently applied the exclusion criteria to finalize the included articles. In cases of disagreement, other authors would participate in the discussion until consensus was reached on whether to include a particular article. Finally, the research team reached a consensus on the final list of articles to be included in the review. The search was conducted until January 2024. For each of the included studies, two separate reviewers extracted the following information: author, year, country, title, aim, study type, sample, methods/scale, and brief summaries/findings.

#### Inclusion criteria

Articles included in this review, regardless of publication date or journal, had to meet the following inclusion criteria: should be peer-reviewed English journal articles; the subjects in the studies must undergo at least one form of empathy measurement; the participants must be medical students; and the research must investigate at least one aspect of the impact of empathy on medical students’ mental health, academic performance, clinical competence, or specialty preference. Both experimental, non-experimental, and mixed-method studies were eligible for inclusion.

#### Exclusion criteria

The exclusion criteria were as follows: editorials, letters to the editor, viewpoints, case presentations, articles not written in English, and those for which we could not obtain the full text. Additionally, articles lacking research methods and references were also excluded.

### Data analysis and presentation

Once the data extraction process was completed, the authors analyzed the study results. The focus of the analysis was to extract data that meted the aim of this integrative review. The first and second authors finished the analysis, which was then reviewed and refined with the assistance of the third author. Once the data evaluation and analysis processes were completed, the review findings were presented in the form of descriptions.

## Results

A total of 2294 articles were returned from PubMed, EBSCO, and Web of Science using search strategies. 1688 articles were remained after deleting duplicates. After removing articles that did not meet the selection criterias and evaluating 3 results that required discussion, 38 results were included in this review. The types of studies included cross-sectional, longitudinal, and network analysis. All research subjects were medical students. Among the 38 articles included in this review, 21 articles used different versions of JSPE/JSE to assess the empathy of medical students [33, 42, 52, 62, 67–75, 85–92], 6 articles used IRI [64, 66, 93–96], 1 article used JSP and IRI [97], 3 article used Toronto Empathy Questionnaire [98–100], 1 article uses JSPE and Toronto Empathy Questionnaire at the same time to evaluate students’ empathy [101], 1 article uses JSPE, QCAE and IRI at the same time to evaluate students’ empathy [65], 1 article used the Basic empathy scale [102], 2 articles used JSPE, IRI and Empathy Quotient [103, 104], 1 article used the interpersonal and communication skills checklist to evaluate the empathy [105], and 1 article evaluated the empathy of medical students by examiners and simulated patient actor [106].

The included researches indicated that empathy might affect the following aspects of medical students:

### Mental Health

A multicenter cross-sectional study used the Thai Mental Health Indicator and the Toronto Empathy Questionnaire to evaluate the mental health and empathy of medical students participating in the experiment, and analyzed the relationship between the two. The results showed that good mental health was related to higher empathy [98]. In order to explore whether the well-being of medical students was related to empathy, a multi-institutional cross-sectional survey used validated tools to measure students’ empathy, burnout and well-being. The results indicated that students’ empathy scores derived from IRI were negatively correlated with burnout and positively correlated with well-being [64]. Their conclusions were consistent with other researches [85, 93, 99, 102]. A study was conducted at medical schools in Spain, and analyzed medical students’ empathy, depression, anxiety and burnout. The researchers found that students who scored higher in the JSE had a lower risk of depression [69]. In order to research the impact of different dimensions of empathy on burnout, Harscher et al. requested medical students to complete the Maslach Burnout Inventory and IRI scale. After statistical analysis, they found that students with high levels of empathic concern had statistically lower scores of burnout over time while students with high levels of personal distress showed statistically higher scores of burnout during medical education [94], similar to another research [95]. And a network analysis measured medical students’ empathy dimensions and depression symptoms using IRI and PHQ-9. A positive correlation was found between depression and personal pain [96]. However, empathic concern and personal pain belong to the affective empathy, and this study did not further explore the impact of cognitive empathy on medical students’ burnout. In another study, researchers measured students’ empathy through JSPE-S and QCAE [65]. Then they chose depression, anxiety and burnout as the indicators of mental health. In this study, positive associations were found between affective empathy and more mental health issues such as depression, anxiety and burnout, while cognitive empathy was the opposite.

### Academic performance

Academic performance reflects medical students’ basic science and clinical knowledge [68]. Austin et al. examined the empathy of medical students from different grades by using JSPE and investigated whether the students’ empathy scores were related to the end-of-year overall academic performance [42]. At any grade, there was no statistical evidence indicated that empathy scores were related to the academic performance of medical students. Moreover, in a cross-sectional study, students’ empathy data was collected using the IRI, and the Spearman Rank Correlation test was used to determine whether or not there was a correlation between students’ empathy and their academic performance. According to the results, academic performance and empathy were not significantly associated [66]. Two studies from Iran [68] and South Korea [86] evaluated the empathy of medical students participating in the studies using the JSE and JSE-S scales respectively, and investigated empathy score correlation with academic performance. Their results, similar to previous studies, showed that there was no significant relationship between empathy among medical students and academic performance.

### Clinical competence

Communication, knowledge, technical skills, and clinical reasoning are all components of clinical competence [107]. Significant relationship between empathy and clinical competence was reported [108]. The authors used JSPE to assess the empathy of medical students. Then they examined the relationship between empathy scores and the global rating of clinical competence assessed by clinical teachers. Their findings indicated a positive relationship between empathy and clinical competence, particularly in the areas of history taking and physical examinations [70]. These results were consistent with previous findings [105]. Ogle et al. assessed the clinical competence of students through the objective structured clinical examination (OSCE). And the empathy of medical students was self-evaluated by the JSPE, and also evaluated by observers. According to results of the t-test, they found that the clinical competence of medical students was positively correlated with the observed empathic behaviors, but not with the self-evaluation empathy score [71]. In another study using OSCE as an evaluation of medical students’ clinical competence, researchers proved that the self-test empathy score from JSPE could predict the communication score of OSCE but not the overall score [67]. In addition, other researchers collected empathy scores of medical students independently evaluated by examiners and simulated patient actors, and compared them with OSCE scores. After statistical analysis, the results showed that students’ empathy scores were positively correlated with the interaction-based part of OSCE, while the skill-based part was almost irrelevant [106]. Wimmers et al. also believed that the relationship between empathy and OSCE might be determined by the interaction with the patient [87].

### Specialty Preference

Empathy has significant impact on medical students’ mental health, clinical competence, and specialty preference. Medical specialties were divided into people-oriented specialties that paid more attention to communication with patients and the technology-oriented specialties that relied more on medical technology [33]. Internal medicine, family practice, pediatrics, neurology, rehabilitation, psychiatry, emergency medicine, obstetrics and gynecology, ophthalmology, and dermatology are all people-focused medical subspecialties. Surgery, radiology, radiation oncology, pathology, and anesthesia are all examples of technology-focused medical subspecialties.

Using self-reported questionnaires, empathy and specialty preferences of medical students were evaluated by Santos et al. They found that compared with students who preferred technology-oriented majors, students with an inclination toward people-oriented specialties typically exhibit more empathy [97]. In another cross-sectional study, researchers collected students’ specialties preference and empathy scores through IRI, JSPE and Empathy Quotient [103]. According to the study, higher scores on empathy were found among students who favored people-focused majors. This conclusion was the same as previous studies [33, 62, 72, 73, 90, 91, 100, 104]. The relationship between empathy and specialty preference has several explanations. However, other researchers put forward different views on the relationship between empathy and medical students’ specialty preferences. In an Iranian study, researchers collected data on medical students’ empathy and specialty preferences through convenient sampling. Two-way ANOVA was computed to assess the difference of empathy and specialty preference, and there was no statistically significant relationship between them, as evidenced by the results [68]. Additionally, Magalhães et al. asked first-year and senior medical students to complete the JSPE to assess their empathy, and then analyzed the differences in JSPE scores between specialty preferences. They hypothesized that students who prefer people-oriented majors could get higher empathy scores. But the results indicated that there was no significant relationship between specialty preference and empathy of medical students [75]. Other studies also reached similar conclusions: empathy could not affect medical students’ specialty preferences [52, 74, 88, 89, 92, 101].

Author, year, country, title, aim, study type, sample, methods/scale and brief summaries/findings of the studies are provided in Table 1 below.

**Table 1:** The methodological characteristics of the research-type studies included in this review

Author, year, and country	Title of the Study	Aim	Study Type	Sample	Methods/Scales	Brief Summaries/Findings
Thomas et al., 2007, USA	How do distress and well-being relate to medical student empathy? A multicenter study	To determine whether lower levels of empathy among a sample of medical students in the United States are associated with personal and professional distress and to explore whether a high degree of personal well-being is associated with higher levels of empathy.	Multi-institutional, Cross-sectional study	1098 medical students	IRI	Empathy scores were negatively correlated with burnout and positively correlated with well-being.
von Harscher et al., 2007, USA	The impact of empathy on burnout in medical students: new findings	To understand the relationship between empathy (Empathic Concern and Personal Distress) and burnout in medical students.	Longitudinal study	353 medical students	MBI, IRI	Students with high levels of empathic concern had statistically lower scores of burnout, while students with high levels of personal distress showed statistically higher scores of burnout.
Carrard et al., 2022, Switzerland	The relationship between medical students’ empathy, mental health, and burnout: A cross-sectional study	To investigate how medical students’ empathy is related to their mental health and burnout.	Cross-sectional study	886 medical students	JSPE, QCAE, IRI, AMSP	Positive associations were found between affective empathy and more mental health issues such as depression, anxiety and burnout, while cognitive empathy was the opposite.
Patricia et al., 2021, Spain	Depression, anxiety, burnout and empathy among Spanish medical students	To conduct a nationwide analysis of the prevalence of mental health problems among medical students.	Multi-center Cross-sectional study	5216 medical students	BDI-II, MBI-SS, STAI, JSE	Students who scored higher in the JSE had a lower risk of depression.
Li et al., 2022, China	The relationship between dimensions of empathy and symptoms of depression among university students during the COVID-19 pandemic: A network analysis	To investigate the nuanced associations between deferent dimensions of empathy and individual symptoms of depression using a network model during the pandemic.	Network analysis	1177 medical students	IRI, PHQ-9	The study showed positive relationships between personal distress and symptoms of depression.
Pitanupong et al., 2023, Thailand	Relationship of mental health and burnout with empathy among medical students in Thailand: A multicenter cross-sectional study	To explore mental health, burnout, and the factors associated with the level of empathy among Thai medical students.	Multicenter Cross-sectional study	336 medical students	Personal and demographic information questionnaire, Thai Mental Health Indicator, MBI, TEQ	The multivariate analysis indicated that mental health was statistically significantly associated with the level of empathy.
Sathaporn et al., 2021, Thailand	The Relationship between Mental Health with the Level of Empathy Among Medical Students in Southern Thailand: A University-Based Cross-Sectional Study	To determine the level of and factors associated with empathy among medical students.	Cross-sectional study	1010 medical students	TEQ, Thai Mental Health Indicator-15	High empathy was a positive factor for mental health.
Brazeau et al., 2010, USA	Relationships Between Medical Student Burnout, Empathy, and Professionalism Climate	To explore relationships between burnout and professionalism using validated instruments.	Cross-sectional study	127 medical students	MBI, JSPE-S, PCI	Scores indicative of higher medical student burnout were associated with lower medical student empathy scores.
Sulaiman et al., 2023, Qatar	Experiences of burnout, anxiety, and empathy among health profession students in Qatar University during the COVID-19 pandemic: a cross-sectional study	To evaluates the prevalence of burnout and its relationship to anxiety and empathy during the COVID-19 pandemic among health profession students in the main governmental institution in Doha, Qatar using validated instruments.	Cross-sectional study	272 medical students	GAD-7, MBI-GS, IRI	Burnout was positively associated with empathy.
Paro et al., 2014, Brazil	Empathy among Medical Students: Is There a Relation with Quality of Life and Burnout?	To assess medical students’ empathy and its associations with gender, stage of medical school, quality of life and burnout.	Cross-sectional, multi-centric study	1350 medical students	MBI-HSS, WHOQOL-BREF, IRI	The empathy scores were moderately correlated with burnout.
Wu et al., 2022, China	Empathy alleviates the learning burnout of medical college students through enhancing resilience	To investigate the relationship between empathy and learning burnout, as well as the mediation effect of resilience in this relation.	Cross-sectional study	588 medical students	Basic empathy scale, Connor-Davidson Resilience Scale, Learning Burnout Scale	Medical students’ empathy ability and their levels of learning burnout were negatively correlated.
Austin et al., 2007, UK	A preliminary study of empathy, emotional intelligence and examination performance in MBChB students	To compare empathy levels in medical students in Years 2, 3 (pre-clinical) and 5 (clinical), to examine gender differences in empathy and EI, and to investigate whether EI and empathy are related to academic success.	Cross-sectional and Longitudinal study	273 medical students	JSPE	There was no relationship between empathy scores and academic performance of medical students.
Jung et al., 2022, Korea	Correlation between medical student empathy and a Korean nationwide comprehensive clinical assessment score at a medical school in Korea	To determine the relationship between JSE-S and clinical comprehensive assessment scores.	Cross-sectional study	108 medical students	JSE-S	Academic performance and empathy were not significantly associated.
Hojat et al., 2002, USA	Empathy in medical students as related to academic performance, clinical competence and gender	To test two hypotheses: firstly, that medical students with higher empathy scores would obtain higher ratings of clinical competence in core clinical clerkships; and secondly, that women would obtain higher empathy scores than men.	Longitudinal study	371 medical students	JSPE	Empathy scores were associated with ratings of clinical competence and gender, but not with performance in objective examinations.
Colliver et al., 1998, USA	Assessment of empathy in a standardized-patient examination	To determine the extent to which 4th-year medical students were checked “empathic” by standardized patients on a performance-based examination, to evaluate the psychometric properties of this simple empathy measure, and to see whether empathy was related to clinical performance on history taking and physical examination.	Cross-sectional study	1048 medical students	interpersonal-and communication-skills checklist	Empathy was an enabling factor in clinical competence, the empathy measure was related to history-taking and physical-examination performance.
Ogle et al., 2013, Australia	Empathy is related to clinical competence in medical care	To investigate the relationship between empathy and clinical competence among medical students.	Cross-sectional study	57 medical students	REM, JSPE-S, OSCE	Observable empathy was strongly associated with medical students’ clinical competence. Self-rated empathy, however, was not associated with clinical competence.
Casas et al., 2017, USA	Associations of medical student empathy with clinical competence	To examine if self-reported empathy in medical students was associated with clinical competence.	Cross-sectional study	590 medical students	JSPE, OSCE	JSPE scores were positively associated with OSCE communication scores in medical students.
Wright et al., 2014, USA	Examiner and simulated patient ratings of empathy in medical student final year clinical examination: are they useful?	To evaluate the empathy scores of examiners and simulated patients in the clinical examination of medical students in the final year.	Cross-sectional study	133 medical students	OSCE	Empathy scores show significant correlation with the interaction based OSCE stations and virtually no correlation with the skills-based stations.
Benabbas 2016, Iran	Empathy in Iranian medical students: A comparison by age, gender, academic performance and specialty preferences	To investigate empathy among Iranian medical students and the possible differences between students of different levels of medical education.	Cross-sectional study	459 medical students	JSE	No statistically significant correlation was found between empathy score and academic performance or specialty of choice.
Javaeed et al., 2022, Pakistan	Empathy scores amongst undergraduate medical students and its correlation to their academic performance	To assess the relationship between empathy and gender, and the academic performance of undergraduate medical students of Azad Kashmir.	Cross-sectional study	151 medical students	IRI	No significant correlation was found between the empathy scores and academic performance.
Wimmers et al., 2010, USA	Assessing medical students’ empathy and attitudes towards patient-centered care with an existing clinical performance exam (OSCE)	To study the ability of OSCE to capture the degree of students’ patient-centeredness and empathy as measured by the PPOS and the JSE.	Cross-sectional study	101 medical students	OSCE, PPOS, JSE	Students’ level of empathy had a moderate association with students’ score on the patient-provider interaction component of the OSCE.
Hussain et al., 2020, Pakistan	Empathy among Students of a Public-Sector Medical University: A Cross-Sectional Study	To determine the empathy among medical students of Pakistan.	Cross-sectional study	273 medical students	JSE-S	The results provided a slight difference in empathy score of students opting for different specialty choices, but it was not statistically significant.
Guilera et al., 2018, Spain	Empathy and specialty preference in medical students. Follow-up study and feedback	To explore the evolution of empathy among medical students based on gender and specialty preferences using several validated scales.	Cross-sectional study	151 medical students	JSPE, IRI, EQ,	Students who preferred people-oriented specialties score higher empathy scores in JSPE and EQ.
Kötter et al., 2021, Germany	The Development of Empathy and Associated Factors during Medical Education: A Longitudinal Study	To investigate the development of empathy during medical education and assessed potential predictors of empathy at different time points in the course of medical studies.	Longitudinal study	43 medical students	JSE-S, HADS, Perceived Medical School Stress scale	A preference for a people-oriented specialty was associated with a higher JSE-S sum score. HADS depression was negatively correlated with JSE-S empathy score.
Stefanović et al., 2015, Serbia	EMPATHY PREDICTING CAREER CHOICE IN FUTURE PHYSICIANS	To address the differences in empathy in the context of career decision making by future physicians.	Cross-sectional study	363 medical students	TEQ	The empathy had a positive effect on choice of people-oriented medical disciplines.
Akgün et al., 2020, Turkey	Medical Students’ Empathy Level Differences by Medical Year, Gender, and Specialty Interest in Akdeniz University	To evaluate the level of empathy among medical students in all years of medical training using two different instruments: the Jefferson Scale of Physician Empathy and the Toronto Empathy Questionnaire.	Cross-sectional descriptive study	300 medical students	JSPE-S, TEQ	Students intending to follow the clinical specialty had higher empathy scores. However, the score difference between specialties was not statistically significant.
Magalhães et al., 2011, Portugal	Empathy in senior year and first year medical students: a cross-sectional study	To study empathy in senior year and first year medical students and the relationship between empathy, gender, and specialty preference.	Cross-sectional study	476 medical students	JSPE-spv	Significant differences in empathy were not found between the students who prefer people-oriented specialties compared to those who favor the technology-oriented specialties.
Mostafa et al., 2014, Bangladesh	Empathy in Undergraduate Medical Students of Bangladesh: Psychometric Analysis and Differences by Gender, Academic Year, and Specialty Preferences	To measure and examine empathy among a sample of undergraduate medical students of Bangladesh.	Cross-sectional study	426 medical students	JSE-S	A nonsignificant difference was found between empathy scores and specialty preferences.
Tavakol et al., 2011, UK	Empathy in UK medical students: differences by gender, medical year and specialty interest	To explore the relationship between undergraduate medical students’ empathy scores relevant to gender, medical school year and future career ambitions.	Cross-sectional study	853 medical students	JSPE	Those students preferring people-oriented specialties would score higher on the empathy scale than students choosing technology-oriented specialties.
O’Tuathaigh et al., 2019, Ireland	Medical students’ empathy and attitudes towards professionalism: Relationship with personality, specialty preference and medical programme	To study how empathy, personality, and background factors might impact on students’ attitudes towards professionalism in medicine.	Cross-sectional study	241 medical students	JSE; NEO-FFI-3; Attitudes towards Professionalism Scale	Empathy did not vary according to career specialty preference.
Chen et al., 2007, USA	A Cross-sectional Measurement of Medical Student Empathy	To measure and examine student empathy across medical school years.	Cross-sectional study	658 medical students	JSPE-S	Students preferring people-oriented specialties had higher empathy scores than students preferring technology-oriented specialties.
Santos et al., 2016, Brazil	Empathy differences by gender and specialty preference in medical students: a study in Brazil	To assess medical students’ empathy and to examine empathy differences by students’ socio-demographic characteristics, including gender, and specialty preference	Cross-sectional and descriptive study	226 medical students	JSPE, IRI	Medical students who intended to pursue a people-oriented profession had higher empathy.
Guilera et al., 2019, Spain	Empathy and big five personality model in medical students and its relationship to gender and specialty preference: a cross-sectional study	To explore the relationship between empathy and personality, and taking into account gender and specialty preference.	Cross-sectional study	110 medical students	JSPE, IRI, EQ, NEO-FFI	Higher scores on empathy were found among students who favored people-focused majors.
Chen et al., 2012, USA	Characterizing changes in student empathy throughout medical school	To examine the trend of empathy longitudinally; determined differences in empathy according to gender and medical specialty preferences.	Longitudinal study	1162 medical students	JSPE-S	Students preferring technology-oriented specialties had lower empathy scores.
Hojat et al., 2005, USA	Empathy in medical students as related to specialty interest, personality, and perceptions of mother and father	To examine relationships between empathy, specialty interest, personality and perceptions of mother and father.	Cross-sectional study	422 medical students	JSPE	Higher scores on empathy were found among students who favored people-focused majors.
Assing et al., 2022, Denmark	A cross-sectional study of student empathy across four medical schools in Denmark-associations between empathy level and age, sex, specialty preferences and motivation.	To examine the associations between empathy scores among Danish medical students and medical school, year of curriculum, age, sex, co-habitation, and parental status, specialty preferences and motivations for choosing medicine as a future profession.	Cross-sectional study	672 medical students	JSE-S	The positive associations were found between empathy scores and specialty preferences for psychiatry and general practice and altruistic motivations for choosing to enroll.
Shashikumar et al., 2014, India	Cross sectional assessment of empathy among undergraduates from a medical college	To assess the empathy ability of medical college undergraduates.	Cross-sectional study	488 medical students	JSPE-S	The relation of mean empathy scores and choice of specialty is inconclusive.
Hasan et al., 2013, Kuwait	Level of empathy among medical students in Kuwait University, Kuwait	To evaluate the level of empathy among medical students in Kuwait University Medical School and its association with sociodemographic factors, stress levels and personality.	Cross-sectional study	264 medical students	JSPE-S, ZKPQ, PSS	Empathy could not affect medical students’ specialty preferences.

**Legend: **

AMSP—Ability to Modify Self-Presentation Scale; BDI-II—Beck Depression Inventory Test; BEES—Balanced Emotional Empathy Scale; BPI-SF—the Brief Pain Inventory Short Form; CARE— Consultation and Relational Empathy Scale; CAD-R—the Pain Coping Questionnaire; DPPRQ-10—Difficult Physician-Patient Relationship Questionnaire; EQ-5D—the EuroQol-5D; F—female; GAD-7—Generalized Anxiety Disorder 7-item scale; HADS-D—Hospital Anxiety and Depression Scale in the German language; IPQ—brief Illness Perception Questionnaire; IRI—the Interpersonal Reactivity Index; IRI-MS—Interpersonal Reactivity Index for Medical Students; JSE—Jefferson Scale of Empathy; JSPE—Jefferson Scale of Physician Empathy; JSPE-S—The Jefferson Scale of Physician Empathy–Student Version; JSPPPE—Jefferson Scale of Patient Perceptions of Physician Empathy; LOT-R— the Life Orientation Test-Revised; M—male; MBI—Maslach Burnout Inventory; MBI-GS(S)—Maslach Burnout Inventory-General Students Survey; MBI-MS—Maslach Burnout Inventory for Medical Students; MBI-SS—Maslach Burnout Inventory Survey for Students; MBI-HSS—Maslach Burnout Inventory – Human Services Survey; OSCE—objective structured clinical examination; PDRQ-15—Patient-Doctor Relationship Questionnaire; PHQ-9—Patient Health Questionnaire-9; PSS—Perceived Stress Scale; PMSS-D—Perceived Medical School Stress scale; PPOS—Patient-Practitioner Orientation Scale; QCAE—Questionnaire of Cognitive and Affective Empathy; REM—Assessment of Empathic Communication in Medical Interviews; RIPLS—Readiness for Interprofessional Learning Scale; STAI—State-Trait Anxiety Inventory; TEQ—Toronto Empathy Questionnaire; WHOQOL-BREF—World Health Organization Quality of Life Assessment; ZKPQ—Zuckerman-Kuhlman Personality Questionnaire.

## Discussion

### Main findings

This integrative review aimed to synthesize the comprehensive impact of empathy on medical students. The results indicated that medical students who scored higher on self-reported empathy scales often have better mental health. However, low level of cognitive empathy and high level of affective empathy might lead to poor mental health among medical students, manifested as depression, anxiety, and burnout. Although there was no correlation between medical students’ empathy and their academic achievement, a positive correlation was observed between empathy and clinical competence, particularly in terms of communication skills. Additionally, most researches indicated that medical students with high empathy tended to choose people-oriented specialties, while students with lower empathy scores tended to choose technology-focused specialties.

### Mental health, academic performance and clinical competence, and specialty preference

The poor mental health caused by high affective empathy and low cognitive empathy could be explained by the guilt and shame caused by both [109, 110]. The term “mental health” refers to a state of well-being that enables individuals to cope with life’s challenges. However, the risk of depression [111], anxiety [112] and burnout [113] was found to be especially high among medical students. Their mental health was worse than general population [112]. Too much or too little empathy may have a negative impact on the mental health of students in medical school. It is evident that this negative effect is driven by high levels of affective empathy and low levels of cognitive empathy. Medical students usually have higher academic workload than those without medical background; they also often have higher exposure to patient pain, fewer social connections, and get less sleep compared to others. As a result, there are some serious challenges that medical students face when it comes to their mental health. A reasonable explanation for the relationship between empathy and clinical competence is that empathy is less relevant to physical exam skills but strongly correlates with communication skills. The positive connection between empathy and clinical competence is mainly determined by the communication. However, there exists a dispute among researchers regarding whether empathy is associated with other aspects of clinical competence. Furthermore, medical students’ specialty preferences may be influenced by interpersonal skills, which are reflected in empathy. The nature of people-oriented specialties means that their graduates have more opportunities to practice empathy than those who choose to focus on technology [114], while technology-oriented majors typically involve a relatively low degree of interpersonal contact [115]. Other studies indicated that the difference in empathy scores between specialties was not statistically significant [88, 89]. This may be because, when choosing their specialties, medical students consider factors such as popularity, income, and night shifts rather than personal empathy.

### Limitations

There are limitations within this review which need to be acknowledged. Firstly, since this review focuses solely on published researches, it may overlook studies implemented by medical educators but not yet published in the literature. Despite rigorous search methodologies, there is a possibility that studies were missed due to the nature of the search strings used, particularly if keywords were not present in the title or abstract. Secondly, empathy is typically measured through self-report questionnaires, which may introduce bias in the results. Volunteers’ self-reported empathy may not accurately reflect their actual levels of empathy. Furthermore, differences in questionnaire usage for the same experiment may yield completely different results, an issue that is not addressed in this review. Additionally, articles written in languages other than English were excluded, potentially excluding relevant studies. This review did not use educational theories, models, and/or frameworks related to the research topic, which resulted in a lack of benefits from theory being a major limitation of the study.

### Recommendations & future research directions

Considering the severe mental health challenges faced by medical students, any method that may help them maintain good mental health state is worth applying. In light of previous research findings, medical educators should develop feasible training programs or courses, such as patient narrative and creative arts, to help medical students reduce affective empathy and improve cognitive empathy within an appropriate range. Ultimately, medical students can maintain good mental health state to cope with challenges in their work and life. Medical educators should attach importance to the positive correlation between empathy and communication skills in clinical competence among students. Teachers should help medical students enhance their communication skills by improving their empathy, ultimately achieving improvement in clinical competence. Due to the lack of specific research on the impact of different dimensions of empathy on clinical competence, this is a meaningful research direction for researchers in this field. In the future, medical educators may effectively help medical students by using the conclusions drawn from research on the relationships between empathy and specialty preferences. For instance, freshmen in medical schools can be assessed their empathy levels to predict their specialty preferences, enabling educators to tailor targeted courses to enhance their competence in their preferred specialties. Furthermore, these conclusions can assist medical educators in identifying students with relatively low empathy scores who prefer technology-oriented specialties, allowing them to provide additional courses aimed at enhancing and maintaining empathy. Given the current scarcity of research on the impact of affective empathy and cognitive empathy on medical students’ specialty preference, further studies in this area are needed. Finally, it is important to note that scores on empathy scales may not accurately reflect medical students’ actual empathy. Future research can evaluate empathy by observing actual empathy behaviors.

## Conclusions

Medical students who score higher on self-reported empathy scales often have better mental health. However, interestingly, positive associations were discovered between affective empathy and mental health issues such as depression, anxiety, and burnout, whereas cognitive empathy presented an opposite trend. There is no correlation between the empathy of medical students and their academic achievements, but it is positively correlated with their clinical competence, especially their communication skills. Moreover, medical students with high empathy tend to choose specialties that are dedicated to people. Because empathy consists of both cognitive and affective components, it has a complex impact on medical students. The findings from the studies reviewed can assist medical educators in their curriculum development efforts. Consequently, it is imperative to develop targeted courses and training for medical students to foster their empathy and reap its benefits.

### Electronic supplementary material

Below is the link to the electronic supplementary material.

Supplementary Material 1

## Data Availability

The datasets used and analyzed during the current study available from the corresponding author on reasonable request.
